# Estimation of genetic parameters and genotype-by-environment interactions related to acute ammonia stress in Pacific white shrimp (*Litopenaeus vannamei*) juveniles at two different salinity levels

**DOI:** 10.1371/journal.pone.0173835

**Published:** 2017-03-22

**Authors:** Xia Lu, Sheng Luan, Baoxiang Cao, Xianhong Meng, Juan Sui, Ping Dai, Kun Luo, Xiaoli Shi, Dengchun Hao, Guomin Han, Jie Kong

**Affiliations:** 1 Key Laboratory of Sustainable Utilization of Marine Fisheries Resources, Ministry of Agriculture, Yellow Sea Fisheries Research Institute, Chinese Academy of Fishery Sciences, Qingdao, China; 2 Laboratory for Marine Fisheries Science and Food Production Processes, Qingdao National Laboratory for Marine Science and Technology, Qingdao, China; 3 College of Fisheries and Life Science, Shanghai Ocean University, Shanghai, China; 4 College of Ocean, Agricultural University of Hebei, University, Huanghua, China; Xiamen University, CHINA

## Abstract

Regarding the practical farming of *Litopenaeus vannamei*, the deterioration of water quality from intensive culture systems and environmental pollution is a common but troublesome problem in the cultivation of this species. The toxicities that result from deteriorating water quality, such as that from ammonia stress, have lethal effects on juvenile shrimp and can increase their susceptibility to pathogens. The toxicity of ammonia plays an important role in the frequently high mortality during the early stage on shrimp farms. However, little information is available regarding the genetic parameters of the ammonia tolerance of juveniles in the early stage, but this information is necessary to understand the potential for the genetic improvement of this trait. Considering the euryhalinity of *L*. *vannamei* and the fact that low salinity can increase the toxicity of ammonia stress, we estimated the heritability of ammonia tolerance in juveniles in 30‰ (normal) and 5‰ (low) salinity in this study using the survival time (ST) at individual level and the survival status at the half-lethal time (SS_50_) at the family level. In the normal and low salinity conditions and for the merged data, the heritability estimates of the ST (0.784±0.070, 0.575±0.068, and 0.517±0.058, respectively) and SS_50_ (0.402±0.061, 0.216±0.050, and 0.264±0.050, respectively) were all significantly greater than zero, which indicates that the ammonia-tolerance of shrimp can be greatly improved. So it might provide an alternative method to reduce mortality, help to enhance resistance to pathogens and reduce the occurrence of infectious diseases. The significant positive genetic correlation between ST and body length suggested that ammonia is more toxic to shrimp in the early stage. The medium-strength genetic correlations of the ST and SS_50_ between the two environments (0.394±0.097 and 0.377±0.098, respectively) indicate a strong genotype-by-environment (G×E) interaction for ammonia tolerance between the different salinity levels. Therefore, salinity-specific breeding programs for ammonia tolerance in shrimp should be purposefully implemented.

## Introduction

The Pacific white shrimp *Litopenaeus vannamei* is an important worldwide aquaculture species [1, 2]. Due to the advantages of euryhalinity, rapid growth, and a high survival rate at high density, *L*. *vannamei* is becoming the most popular cultured species in the world and generally uses semi-intensive and intensive culture modes [3]. However, these culture practices often lead to the degradation of the water quality due to uneaten food and the waste products of the animals. Additionally, the vagaries of climates and the serious pollution of aquaculture environments jointly cause the quality of the culture water to decrease. The toxic factors associated with deteriorating water quality exhibit very strong correlations with the high mortality of *L*. *vannamei* in farms [4–6].

High concentrations of ammonia are the most common and greatest toxic factor in the deteriorating water quality, which does serious harm to crustaceans, mollusks and fish [4, 7–9]. Much research has been performed to detect the detrimental effects of ammonia to shrimp, and this research has revealed that the accumulation of ammonia in pond water reduces growth, damages the hepatopancreas and gills, increases oxygen consumption, decreases the osmoregulatory capacity, reduces the ability of the hemolymph to transport oxygen, affects the molting frequency, and even causes high mortality in shrimp [10–18]. More important than all of the above, increased ammonia in the water can suppress the immune defense system of shrimp and increase their sensitivity to pathogens [19–21]. We previously performed a comparative transcriptome analysis to understand the molecular mechanisms of the detrimental effects of ammonia stress in *L*. *vannamei*, and we found that the majority of the key genes involved in the response to ammonia stress are potentially involved in immune defense function [22].

Because the aquaculture environment is becoming worse, and ammonia stress causes grievous harm to shrimp aquaculture, it is necessary to seek possibilities for culturing shrimp that efficiently tolerate ammonia stress, which might represent alternative methods for reducing mortality and infectious diseases. Selective breeding programs are an effective method to gain genetic improvements and have been utilized to improve performance in terms of growth, survival, reproduction, resistances to viruses and adverse environmental stress in shrimp [23–27]. Knowledge about the heritability of ammonia tolerance in shrimp is a prerequisite to understanding the potential for the genetic improvement of this trait. Previous studies have revealed that ammonia stress is more toxic to the earlier ontogenetic development of aquaculture organisms [28–30], and it has been widely found to exert lethal effects on penaeid shrimp juveniles [31–37]. The shrimp in the early juvenile stage are critical for successful breeding because high rates of mortality often occur during this period. We have performed an investigation to estimate the heritability of ammonia resistance in shrimp at the stage with an average body weight of 3.3 g in the normal salinity (30‰) [38], but there are no reports regarding the genetic parameters of ammonia tolerance in juvenile shrimp in the early stage. Additionally, the phenotype of a quantitative trait is determined by genetic and environmental resources and their interactions [39]; thus, genotype-by-environment (G×E) interactions will play an important role in genetic improvement [40]. *L*. *vannamei* is cultured at different salinity levels due to its euryhalinity. However, many studies have revealed that low salinity can increase the toxicity of ammonia to the shrimp [34, 35, 41–43], and we have also verified that ammonia stress can influence the pathway that is involved in osmoregulation [22]. If the G×E interaction for ammonia tolerance is significant at different salinity levels, the selection response for this trait will vary across different salinity levels [44]. Based on the above reasoning, it is necessary to understand the heritability of ammonia tolerance in the early stage and the G×E interactions at different salinity levels in *L*. *vannamei*.

Considering that the majority of the production of *L*. *vannamei* occurs in seawater with normal salinity and freshwater with low salinity, in the present study, we estimated the heritability of ammonia tolerance in *L*. *vannamei* juveniles in the early stage (average body weight, 0.5 g) at two different salinity levels (30‰ and 5‰) and estimated the genetic correlations between body length and ammonia tolerance within the two environments to verify the relationship between ammonia tolerance and ontogenetic development. Additionally, in the present study, we also detected the G×E interaction between the two salinity levels. Our goal was to understand the potential for the genetic improvement of ammonia tolerance in *L*. *vannamei* in the early stage in normal and low salinity because such improvement might represent an alternative method for reducing the mortality of *L*. *vannamei* and will also be helpful for enhancing resistance to pathogen and reducing infectious diseases.

## Materials and methods

### Origin of the base population and selection procedure

The program was conducted at a Hebei Xinhai Aquatic Biological Technology Co., Ltd. Facility located in the Huanghua (longitude 111.3366, latitude 38.37738), Hebei province, China. The founders were collected from seven improved commercial strains that were introduced from different companies in the United States and Singapore. The virus-free individuals from the seven strains were tagged using numbered rings that were placed on one ocular peduncle and used to produce the base population (G_0_) via an incomplete diallel cross experiment. The G_0_ was constructed with 130 full-sib families using artificial insemination with 114 males and 108 females in 2011 and included 69 half-sib families. Individual estimated breeding values (EBV) for body weight and the family EBV for survival rate from the test were weighted into the selection index and used to rank the breeding candidates as described by Luan et al. [45]. The families with low selection indices (<100) were eliminated from the breeding program, and the males and females with high selection indices (>100) from the remaining families were selected to produce the next generation. During the implementation process of the present program, the selection population was closed, and the generations were discrete. The pedigree of the selection population is clear and forms a relationship matrix. The shrimp production scheme was provided in our previous study [38].

The present experimental shrimp were from 91 full-sib families (52 half-sib families) of the fifth generation (G_5_). The half-sib families were produced by two females mating with one male or two males mating with one female, and all of the families were produced by artificial insemination. The processes of family construction, hatching, and larvae rearing were described in detail in our previous study [46]. At the post-larvae stage of approximately 45 days, approximately 400 post-larvae were randomly selected from each family and equally transferred into tow net cages (0.5 m^3^) that were separately fixed in two large ponds (60 m^3^) for separate rearing at the same density for each family. The seawater in one of the ponds was diluted with filtered fresh water from 30‰ to 5‰, which lasted for approximately three weeks. However, the seawater in the other pond was maintained at 30‰. Aside from the salinity, the other management conditions were maintained identically between the two ponds.

### Challenge with high concentration ammonia

To acquire the individual survival times for the estimations of the genetic parameters, we performed a pre-experiment to obtain the proper concentrations of ammonia in the 30‰ (normal) and 5‰ (low) salinity conditions. Based on the results of our pre-experiment, the concentrations of ammonia required to kill all of the shrimp after 72-h challenges were 32 mg/L and 18 mg/L in the normal and low salinity conditions, respectively. The ammonia solution was prepared using NH_4_Cl (Aldrich, Milwaukee, WI, USA).

The juveniles were too small (the average body weight of 0.50 g and average length of 3.73 cm) that they could not be tagged with visible implant elastomer (VIE) when the experiment began. Therefore, forty individuals were randomly selected from each net cage (each family) and transferred to separate 100 L tanks filled with 35 L 30‰ or 5‰ salinity water for ammonia stress tests under the same conditions of density and environmental management. After a period of three days for a temporary rearing, the ammonia acute stress experiment was initiated. During the experiment, the shrimp were given feedstuff twice per day, and the management environments were maintained identically between the tanks. The dissolved oxygen level was no less than 6 mg L^-1^, the pH ranged from 8.00 to 8.06, and the temperature was approximately 27±0.5℃. The tanks were cleaned daily by suction to remove feces, and the water that was removed by suction was replaced with clean water with the same concentration of ammonia. The dead shrimp were collected and recorded every hour during the experiment. When the shrimp sank, stopped movement and lost responsiveness to external stimulation, they were defined as dead and removed from the tanks with a scoop net. The family ID and death time of each dead shrimp were recorded to quantify the individual survival times (STs) and survival statuses (1 = alive, 0 = dead) at the half lethal time (SS_50_). Because the animals were too small that body weight measures would introduce large measurement errors due to the different degrees of excess water on the shrimp, we selected body length (BL) as an index of growth. Therefore, the individual BLs were also recorded when the shrimp died, which enabled us to investigate the relationship between ammonia tolerance and growth stage.

### Statistical analysis

#### Variance components and heritability estimates

An analysis of the descriptive statistics was conducted using the MEANS procedure [47]. The complete pedigree of this breeding program from G_0_ to G_5_ was used in the following analysis to account for the genetic relationships among the individuals. The common environmental effect was omitted in this analysis due to the separate rearing of the families during the experiment. The ammonia tolerance was defined by the ST (individual level) and the SS_50_ (family level) in the present study. The variance components and heritabilities were calculated for ST and SS_50_ using the data from the normal and low salinity conditions and the merged data from the two conditions. Therefore, these variables are denoted as the ST_H_/SS_50H_, ST_L_/SS_50L_, and ST_M_/SS_50M_ for the normal salinity, low salinity, and merged data, respectively.

The individual animal model was used to estimate the heritability of the ST in the shrimp that were challenged with the high concentration of ammonia. Body length exhibited a linear relationship with ST (*P* < 0.01) and was fit as a covariate in the model. The fitted model was as follows:

$$Y_{ijk}=\mu {+\text{Salt}}_{i}+b\times BL_{j}+a_{k}+e_{ijk}$$ (Model 1)

where *y*_*ijk*_ is the observed ST of the *k*^th^ individual, *μ* is the overall mean, Salt_*i*_ is the fixed effect of the *i*^th^ salt level (two levels) and it was not contained when estimated for the separate environments, BL_*j*_ is the covariate of the *j*^th^ body length, *b* is the regression coefficient, *a*_*k*_ is the random additive genetic effect of the *k*^th^ individual, and *e*_*ijk*_ is the random residual effect associated with observation *ijk*. The phenotypic variance ($\sigma_{p}^{2}$) was taken as the sum of all of the variance components as follows: $\sigma_{p}^{2}=\sigma_{a}^{2}+\sigma_{e}^{2}$. The heritability (*h*^2^) was calculated as the ratio between the genetic variance and the total phenotypic variance ($h^{2}=\sigma_{a}^{2}/\sigma_{p}^{2}$).

A standard threshold (probit) and a sire–dam model were used in this study to estimate the heritability of the SS_50_. The model was written in ASReml as follows [48]:

$$\lambda_{ijkl}=\mu +{\mathrm{Salt}}_{i}+b\times BL_{j}+{\mathrm{Sire}}_{k}+{\mathrm{Dam}}_{l}$$ (Model 2)

$$y_{ijkl}=\left\{ \begin{array}{c} \;0\;\mathrm{if}\;\lambda_{ijkl}\leq 0\\ 1\;\mathrm{if}\;\lambda_{ijkl}>0 \end{array}\right.$$

where *y*_*ijkl*_ is the survival status (1 = alive, 0 = dead) of the *j*^th^ shrimp, *λ*_*ijkl*_ is the underlying liability of *y*_*ijkl*_, which was assumed to be a cumulative standard normal distribution, *μ* is the overall mean, BL_*j*_ is the covariate of the *j*^th^ body length, *b* is the regression coefficient, and Sire_*k*_ and Dam_*l*_ are the additive genetic effects of the *k*^th^ sire and the *l*^th^ dam, Sire or Dam ~(0, $A\sigma_{sd}^{2}$) ($\sigma_{sd}^{2}=\sigma_{s}^{2}=\sigma_{d}^{2}$), where *A* is the additive genetic relationship matrix among all shrimp. The residual variance of *λ* was assumed to be 1. The phenotypic variance was the sum of $2\sigma_{sd}^{2}$ and $\sigma_{e}^{2}$: $\sigma_{p}^{2}=2\sigma_{sd}^{2}+\sigma_{e}^{2}$. Heritability was computed as the ratio between $4\sigma_{sd}^{2}$ and $\sigma_{p}^{2}$ ($h^{2}=4\sigma_{sd}^{2}/\sigma_{p}^{2}$). The heritability would be overestimated in this model, so the estimates for SS_50_ then were adjusted according to Robertson and Lerner [49].

The estimation of the heritability of BL was performed using the ASReml package [48]. The individual animal model was used to estimate the genetic parameters for the BLs of the shrimps that were challenged with high concentrations ammonia. The age at the end of the experiment exhibited a linear relationship with body length and was fit as a covariate in the model. All of the fixed effects and the covariates were statistically significant (*P* < 0.01). The fitted model was as follows:

$$y_{ijk}=\mu {+\text{Salt}}_{i}+b\times {\mathrm{Age}}_{j}+a_{k}+e_{ijk}$$ (Model 3)

where *y*_*ijk*_ is the observed BL of the *k*^th^ individual, *μ* is the overall mean, Salt_*i*_ is the fixed effect of the *i*^th^ salt level (two levels), and it was not contained when estimated for the separate environments, *b* is the regression coefficient, Age_*j*_ is the covariate of the *j*^th^ individual’s age when the experiment ended, *a*_*k*_ is the random additive genetic effect of the *k*^th^ individual, and e_*ijk*_ is the random residual effect associated with observation *ijk*. The phenotypic variance ($\sigma_{p}^{2}$) was the sum of all variance components: $\sigma_{p}^{2}=\sigma_{a}^{2}+\sigma_{e}^{2}$. The heritability was calculated as the ratio between the animals’ genetic variance and the total phenotypic variance as follows: ($h^{2}=\sigma_{a}^{2}/\sigma_{p}^{2}$).

The *z*-scores were used to test whether the heritability estimates were significantly different from each other and whether the heritability estimates were significantly different from zero [50].

$$Z=\frac{x_{i}-x_{j}}{\sqrt{\sigma_{i}^{2}+\sigma_{j}^{2}}}$$

where *x*_*i*_ and *x*_*j*_ are the heritability estimates from different models and different salinity levels, and $\sigma_{i}^{2}$ and $\sigma_{j}^{2}$ are their respective standard errors. Both *x*_*j*_ and $\sigma_{j}^{2}$ were set to be zero when testing whether an estimate was significantly different from zero. The resulting *z*-score was then tested against a large-sample normal distribution.

#### Genotype by environment interaction estimate

The homologous ST, SS_50_, and BL in the different environments were considered to be different traits; thus, the genotype by environment (G×E) interactions were estimated based on the genetic correlations of these traits between environments. The phenotypic (r_p_) and genetic (r_g_) correlations of the ST and BL values between the two salinity environments were estimated using the bivariate animal model described above. Because one animal can only be tested in one environment, there were no environmental (residual) correlations between the homologous traits, and the environmental covariance was set to be zero in the bivariate analysis [51]. Because of the convergence problem, the r_g_ between the two salinity environments for the SS_50_ was calculated with the estimated breeding values. A G×E interaction was measured as the difference between the genetic correlation and 1. Thus, genetic correlations closer to 1 indicated smaller the G×E interactions. The genetic correlations between the environments were used to measure the degree of re-ranking due to G×E interactions. Additionally, the r_p_ and r_g_ values between the STs and BLs within environments were estimated using the bivariate animal model, which is the extension of Model 1 and Model 3, with the ASReml package [48]. The statistical significances of the genetic correlations within environments and between environments were assessed using the confidence intervals (CIs). The CIs of the genetic correlations were calculated using r_g_, the standard errors (SEs) and *Z*_0.025_, which is 1.96 (i.e., [r_g_−1.96×SE, r_g_+1.96×SE]). If 1 or 0 was contained within the CI, the difference between the genetic correlation and 1 or 0 was not significant.

## Results

### Descriptive statistics

**Fig 1:**
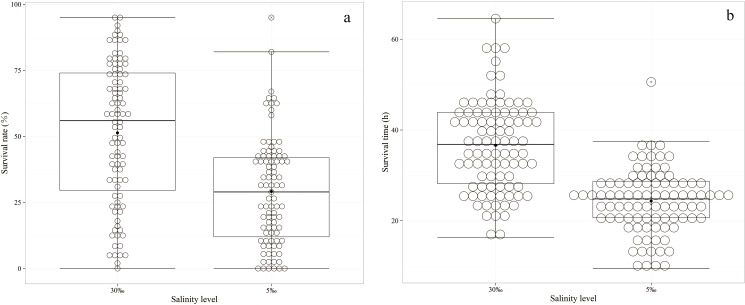
(a) Boxplot of the survival rates of the families at the half-lethal time. (b) Boxplot of the average survival times of the families. The 25^th^ (upper line), median (inside line) and 75^th^ (bottom line) percentiles of the families are plotted as boxes. The minima, maxima, and the observed values are shown as -, -, and ○, respectively.

A total of 7221 individual records, including 3624 records from the normal salinity condition and 3597 records from low salinity condition, were obtained and analyzed in the present study. The number of final testing individuals was less than the total number of starting individuals (7280), which might have been due to cannibalism during the experimental process. The numbers of samples, simple means, minima, maxima, standard deviations and the coefficients of variation for the survival rate of each family at the half-lethal time (SS_50_), ST, and BL are given in Table 1. The 25^th^ percentiles, median percentiles, 75^th^ percentiles, minima, maxima, and outliers of the SS_50_ and average ST values of each family are displayed in Fig 1. The results revealed the SS_50_ values under acute ammonia stress in the normal and low salinity conditions varied substantially between the families, but the variance was higher following exposure to acute ammonia stress in the normal salinity condition (Table 1; Fig 1A). Additionally, the STs under acute ammonia stress in the normal and low salinity conditions also varied substantially between the families and the overall individuals, but the variance was higher when analyzed at the individual level than the family level as indicated by the higher standard deviation (SD) and coefficient of variation (CV). The CVs of the STs were 27.38% and 29.27% among the 91 families in the two environments, but these values were 46.87% and 54.48% among the individuals in the two environments (Table 1). Although the concentration of ammonia (32 mg/L) was higher in the normal salinity condition than in the low salinity (18 mg/L) condition, the average ST under acute ammonia stress in the normal salinity condition (36.64 h) was substantially greater than that in the low salinity condition (24.80 h), and the variance was higher in the normal salinity condition (Table 1; Fig 1B). Regarding the BL, it also exhibited higher variance when analyzed at the individual level than at the family level, but there was no significant difference between the two environments (Table 1).

**Table 1:** Numbers of samples/families (N) and the means, minima, maxima, standard deviations, and coefficients variation of the ST, SR_50_, and BL values.

Traits		N		Mean	Minimum	Maximum	Standard deviation	Coefficient variation (%)
ST (h)	30‰	individuals	3624	36.64	2.00	78.00	16.94	46.23
		families	91	36.64	16.32	64.56	10.03	27.38
	5‰	individuals	3597	24.80	2	69.00	13.51	54.48
		families	91	24.80	9.50	50.58	7.26	29.27
SR_50_ (%)	30‰	families	91	50.78	0	94.87	26.80	52.78
	5‰	families	91	50.27	2.78	95.00	19.29	38.37
BL (cm)	30‰	individuals	3624	3.77	1.00	8.80	0.93	23.85
		families	91	3.77	2.84	4.94	0.42	11.14
	5‰	individuals	3597	3.70	1.00	9.30	0.90	24.32
		families	91	3.70	2.63	4.79	0.40	10.81

**Fig 2:**
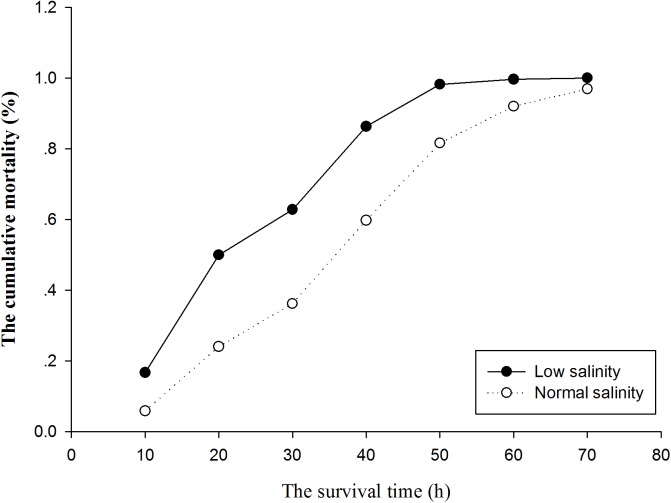
Cumulative mortality of *L*. *vannamei* juveniles during the acute ammonia stress in normal and low salinity conditions.

**Fig 3:**
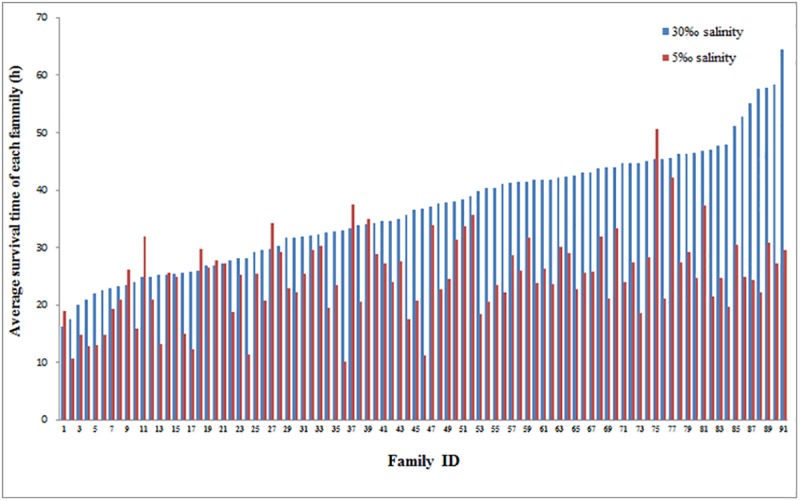
The average survival time for each family of *L*. *vannamei* juveniles during acute ammonia stress in normal and low salinity conditions.

The cumulative mortality and the average survival time of each family under acute ammonia stress in the normal and low salinity conditions are displayed in Fig 2 and Fig 3, respectively. The cumulative mortalities of the families under acute ammonia stress in the low salinity condition were substantially greater than those in the normal salinity condition at each sampling point (Fig 2). All of the individuals in the low salinity condition were dead after 69 h of exposure to acute ammonia stress, but at this time, 115 individuals (3.5%) in the normal salinity condition were still alive. It showed that the average survival time exhibited large variation between the families within the same environment and between the two environments, but the average survival time of the majority of the families under acute ammonia stress in the low salinity condition was substantially lower than that of the families in the normal salinity condition (Fig 3).

### Variance components and heritability estimations

Estimates of the variance components and heritability of ST, SS_50_, and BL created with different models are provided in Table 2. All of the heritability estimates were significantly greater than zero (*P* < 0.01). According to the classification reported by Cardellino and Rovira [52] (i.e., low, 0.05–0.19; medium, 0.20–0.44; high, 0.45–0.64; and very high, >0.65), the heritability estimate of ST_H_ (0.784±0.070) was very high, the heritabilities of ST_L_ (0.575±0.068) and ST_M_ (0.517±0.058) were high, and the heritabilities of SS_50H_ (0.402±0.061), SS_50L_ (0.216±0.050) and SS_50M_ (0.264±0.050) were medium. The heritability estimate of ST_H_ was significantly greater than those of ST_L_ and ST_M_ (*P*<0.05), but there was no significant difference between ST_L_ and ST_M_ (*P*>0.05). The heritability estimates of ST were all significantly greater than that of SS_50_ (*P* < 0.01). The heritability of SS_50H_ was significantly greater than that of SS_50L_ (*P* < 0.05), but there was no significant difference between SS_50H_ and SS_50M_ (*P*>0.05), and there was also no significant difference between SS_50L_ and SS_50M_ (*P*>0.05). The heritability estimates of BL_H_ (0.346±0.052), BL_L_ (0.386±0.054), and BL_M_ (0.291±0.042) were all medium based on the above classification, and the values for BL_H_ and BL_L_ were higher than that of BL_M_ but were not significantly different from each other (*P* > 0.05).

**Table 2:** Variance components and heritabilities of for ST, SS_50_ and BL.

Traits	Variance components			Heritability	Phenotypic correlation	Genetic correlation
	Additive genetic variance	Random residual error variance	Phenotypic variance	*h*^2^±SE		
ST_H_	220.852	60.708	281.560	0.784±0.070^a^	0.286±0.076*	0.394±0.097*
ST_L_	111.258	82.145	193.403	0.575±0.068^b^		
ST_M_	122.199	113.975	236.172	0.517±0.058^b^		
SS_50H_	1.085	1.000	1.724	0.402±0.061^c^	0.273±0.100*	0.377±0.098*
SS_50L_	0.439	1.000	1.293	0.216±0.050^d^		
SS_50M_	0.572	1.000	1.381	0.264±0.050^c^		
BL_H_	0.315	0.596	0.911	0.346±0.052	0.248±0.047*	0.535±0.096*
BL_L_	0.325	0.518	0.843	0.386±0.054		
BL_M_	0.254	0.618	0.871	0.291±0.042		

^a, b, c, d^ represent the significant differences among the traits.

* The estimate is highly significantly different from 0 and 1 (*P*<0.05). H, L, and M represent the variance components, and heritability was calculated using the data from the normal and low salinity conditions as well as the merged data, respectively.

### Genetic corrections within and between environments

To understand the effects of the G×E interactions on the three traits, particularly the trait of ammonia tolerance, genetic correlations were estimated between the two environments, and the results are displayed in Table 2. The phenotypic and genetic correlations among the three traits between the two environments were all positive, and their genetic correlations were higher than their respective phenotypic correlations. The estimated genetic correlation for BL between the two environments was higher (0.535±0.096) than those of ST (0.394±0.097) and SS_50_ (0.377±0.098) (Table 2). The genetic correlations for ST, SS_50_ and BL between the two environments were significantly different from 0 and 1 based on the CIs (ST, 0.20–0.58; SS_50_, 0.19–0.57; and BL, 0.35–0.72). These results indicate a strong re-ranking effect for ammonia tolerance and growth between the two environments.

The phenotypic and genetic correlations within the two environments for ST and BL are given in Table 3. The phenotypic and genetic correlations between the two traits within the two environments were also all positive, and their genetic correlations were greater than their respective phenotypic correlations. The phenotypic and genetic correlations between ST and BL were higher in the normal salinity condition (r_p_ = 0.416±0.017; r_g_ = 0.779±0.037) than in the low salinity condition (r_p_ = 0.298±0.021; r_g_ = 0.568±0.048).

**Table 3:** Correlation analysis based on phenotypic and breeding values between ST and BL.

Traits	Normal salinity		Low salinity	
	ST	BL	ST	BL
**ST**	-	0.779±0.037*	-	0.568±0.048*
**BL**	0.416±0.017*	-	0.298±0.021*	-

* Estimate is highly significantly different from 0 and 1 (*P*<0.05). The phenotypic correlations are under the diagonal, and the genetic correlations are above the diagonal.

## Discussion

In this study, we first reported the heritability of ammonia tolerance (adversity-stress adaptability) for juveniles in the early stage in normal salinity (30‰) and low salinity (5‰) conditions and the G×E interaction between the two environments to explore the possibility of reducing the mortality of shrimp by selection. Many studies have reported that ammonia stress is more toxic to shrimp in early stages [28–30, 53–55], and this finding was corroborated in the present study. In this experiment, approximately 98.6% of the individuals with an average body weight 0.5 g (average body length of 3.7 cm were dead following exposure to 32 mg/L ammonia for 72 h (pH = 8.05, temperature = 26.5±0.5℃, salinity = 30‰). However, all of the *L*. *vannamei* individuals with the an average body weight of 3.3 g were dead following exposure to 63 mg/L ammonia for 218 h (pH = 8.0, temperature = 25℃, salinity = 30‰) [38]. Sun et al. [56] reported that the half-lethal concentration of ammonia is 32.44 mg/L at 72 h (pH = 8.15, temperature = 27℃, salinity = 20‰) for *L*. *vannamei* with an average body length of 5.0 cm. Although high temperatures and low salinities can increase the toxicity of ammonia stress, the shorter survival time at lower concentrations of ammonia in our study also suggests that younger shrimp are more sensitive to ammonia toxicity. Additionally, we also observed that the lower salinity increased the sensitivity of shrimp to the toxic effects of ammonia. The lethal concentration of ammonia at 72 h in the normal salinity condition (30‰) was 32 mg/L, which is significantly higher than the 18 mg/L value observed in the low salinity condition (5‰). The average survival time of all the individuals under acute ammonia stress in the normal salinity condition was 36.61 h, which is substantially greater than the 24.80 h observed in the low salinity condition (Table 1), and the average survival time of majority of the families under acute ammonia stress in the normal salinity condition was significantly greater than that in the low salinity condition (Fig 3). The cumulative mortalities of the families in the normal salinity condition were significantly lower than those in the low salinity condition at each sampling point (Fig 2). The above results indicated that low salinity increased the toxicity of ammonia stress to shrimp, which is consistent with the results of previous studies [34, 35, 41–43]. Our results suggest that the selection of ammonia-tolerant shrimp should be performed in the early stage and in low salinity water.

A high heritability of a trait suggests that rapid and highly accurate genetic improvement can be obtained by selection. Although many studies have been performed to detect ammonia toxicity for aquatic organisms [10–18], very few reports are available regarding the genetic parameters related to ammonia tolerance in aquatic species; thus, there is little information against which to compare our results. Currently, the only previous report of the heritability of ammonia resistance in shrimp with an average body weight of 3.3 g that are subjected to ammonia stress in normal salinity conditions came from our group [38]. In that report, the heritabilities of ST and SS_50_ were 0.154±0.045 and 0.148±0.040, respectively, and these values were not significantly different from each other. In the present study, we estimated the heritability of ammonia tolerance of shrimp with an average body weight of 0.5 g under ammonia stress in normal and low salinity conditions. The heritability estimates of ST (ST_H_ = 0.784±0.070; ST_L_ = 0.575±0.068; ST_L_ = 0.517±0.058) and SS_50_ (SS_50H_ = 0.402±0.061; SS_50L_ = 0.216±0.050; SS_50M_ = 0.264±0.050) under ammonia stress at the two salinity levels were all significantly greater than that reported in the previous study, which indicates that improving the ammonia tolerance of shrimp via selection performed at the early stage could be quite advantageous. Although we did our best to maintain the same environment between the tanks in the present study, the absent of common environmental effects because of the juveniles without communal rearing during the experiment may have contributed to the overestimation of the heritability estimates of ammonia tolerance, because the exclusion of common environmental effects can inflate heritability estimates [57–59]. In our previous study, an ammonia challenge experiment was performed in four tanks, and significant tank effects were detected, so the fixed effect of the tank in the models might have reduced the additive genetic variance to a certain extent, but the absence of common environmental effects also might have resulted in overestimations of the heritability estimates [27, 60]. The higher heritability of ammonia tolerance observed in the present study might have been due to cryptic genetic variation (CGV) that emerged due to ammonia stress, which could have increased the additive genetic variance (*V*_*a*_, i.e., the heritable phenotypic variation). Because shrimp in the early stage are more sensitive to the ammonia stress, the CGV is more easily released. Additional information about CGV can be found in the review by Paaby and Rockman that was published in Nature Reviews Genetics [61]. Genomic matrices based on molecular information provide a more precise method for the estimation of heritability and can thus enhance the accuracy of selective breeding, and this approach has been successfully applied in animal husbandry and agriculture [62, 63]. With the development of molecular biological technology, the cost of obtaining genotypic information from individual shrimp (e.g., through sequencing-based or SNP panel-based genotyping technology) will be substantially reduced, and a few molecular genetic/genomic resources will become publically available. At this time, we will use genomic matrices for heritability estimation, which will enable families to be communal reared without tags.

In the present study, the heritability estimates of ST were all significantly higher than those for SS_50_, which is inconsistent with our previous results that indicated that the heritability estimates of ST and SS_50_ are basically the same [38]. The significantly lower heritability of SS_50_ compared with ST might have resulted from the smaller sample size at the family level (91) for SS_50_ compared to the individual level (above 3500) for ST. Additionally, the heritability estimate of ammonia tolerance at normal salinity was significantly greater than that at low salinity in the present study, which might have resulted from the incomplete release of the additive genetic variance among the individuals and families because the time to death was shorter and more intensive in the low salinity condition; after 20 h of ammonia stress, the cumulative mortality in the low salinity condition was 50%, whereas this value was only 24% in the normal salinity condition (Fig 1). The heritability estimates of ST and SS_50_ from the merged data from the environments were lower than those in the normal salinity condition, which indicates that salinity plays an important role in the estimation of the heritability of ammonia tolerance. After this experiment, a WSSV infection experiment was performed with the ammonia-sensitive and ammonia-tolerant populations to detect the relationship between ammonia tolerance and WSSV resistance. The results revealed that the ammonia-tolerant population also exhibited a significantly greater resistance to WSSV than the ammonia-sensitive population (this result will be published soon in an article entitled “The investigation for the susceptibility difference of ammonia-tolerant and ammonia-sensitive populations to WSSV infection in *Litopenaeus vannamei*”). Fortunately, the ammonia-tolerant shrimp can be improved largely due to high heritability, which might provide an alternative method to reduce mortality, aid the enhancement of resistance to pathogens, and reduce infectious diseases.

Growth is a very important trait in selective breeding programs because it is highly correlated with economic return. The estimates of the heritability of growth in *L*. *vannamei* have primarily focused on harvest body weight [27, 64], and very little information is available regarding juveniles. Although the absence of the common environmental effects could have resulted in the overestimation of the heritability of BL in the present study, the medium heritabilities (0.346±0.052, 0.386±0.054, and 0.291±0.042) in the two environments still indicated that there is potential for improving the growth of juveniles. The growth performances of the families between the two environments were not fully released due to the short communal rearing period (approximately three weeks), and thus, the heritabilities of growth observed in the two salinity levels in the present study are only preliminary results. To understand the difference in growth performance between normal and low salinity conditions, estimates of the heritability of growth in the two salinity levels is carried out in a separate experiment with a longer testing time (approximately three months) in our program, and the results of such an experiment will be published later. Previous studies have reported negative correlations between body weight and resistance traits (such as TSV resistance and WSSV resistance) in *L*. *vanname* [23, 24]. However, a strong positive correlation between body length and ammonia tolerance was detected in the present study, which is consistent with the previous studies [28, 38]. The strong positive correlation between body size and ammonia tolerance suggests that it is necessary to select ammonia-tolerant shrimp during the early stage and that selection high growth will not adversely affect ammonia tolerance performance in shrimp.

G×E interactions refer to genotypes that respond differently in different environments and thus result in different performances [65–67]. Genetic correlations across environments that are significantly different from 1 indicate the existence of re-ranking effects, i.e., genotypes that should be ranked differently in different environments [68]. The results from the simulation study of Sae-Lim et al. [69] suggested that more reliable estimates of the G×E interactions can be obtained with population sizes greater than 2000 (i.e., 50–200 families and 10–40 individuals of each family) and heritabilities greater than 0.30. The above conditions are suitable to the situation in our study, and thus, the present estimates of G×E interactions are credible. Generally, selection for a trait in aquaculture is always performed in the main environment; for example, ammonia tolerance would typically be selected for in normal salinity conditions. However, the offspring of the excellent shrimp procured from selection are transferred to different commercial production environments in most cases. Therefore, strong re-ranking effects may occur and result in different responses to ammonia stress at different salinity levels if there is a strong G×E interaction for this trait, and these effects would influence selection accuracy and the total economic gains. In the present study, we detected a strong re-ranking effect (i.e., a low genetic correlation) for ammonia tolerance between the different salinity levels (Table 3), which suggests that the genetic advantage of ammonia tolerance can be significantly influenced by salinity. Additionally, the genetic correlations across environments would decrease along with increases in differences between environments [70, 71]. When a strong G×E interaction exists for a trait, it is better to perform selection in multiple environments, such as the nucleus environment and other production environments [72]. Therefore, salinity-specific breeding programs for ammonia tolerance in shrimp should be purposefully implemented.

## Conclusion

Overall in this study, we first reported the heritability of ammonia tolerance in juveniles in the early stage and the G×E interaction between the normal and low salinity to explore the potential for improving ammonia tolerance in shrimp by selection. The results revealed that the heritability of ammonia tolerance was medium to high in *L*. *vannamei* juveniles, which suggests that rapid genetic gains in terms of ammonia tolerance could be obtained by increasing the selection intensity in our selective breeding program. However, the heritability of ammonia tolerance in the normal salinity condition was significantly higher than that in the low salinity condition. Additionally, a strong G×E interaction for ammonia tolerance was detected between the normal salinity and low salinity conditions, which suggests that salinity-specific breeding programs for ammonia tolerance in shrimp should be purposefully implemented and that the normal salinity environment is a better choice based on the faster rate of genetic improvement due to higher heritability. Additionally, the significantly and strong positive correction between ammonia tolerance and body size suggests that ammonia-tolerant shrimp should be selected in the early stage.

## Supporting information

S1 Table The heritability of SS_50_ from probit and the formula for adjusting the heritability. (XLSX)

S2 Table The individual data for the analysis in the present study. (XLSX)

S3 Table The original data for Fig 3. (XLSX)
